# Effect of Having a Sense of Purpose in Life on the Risk of Death from Cardiovascular Diseases

**DOI:** 10.2188/jea.JE2007388

**Published:** 2008-10-01

**Authors:** Megumi Koizumi, Hiroshi Ito, Yoshihiro Kaneko, Yutaka Motohashi

**Affiliations:** 1 Department of Public Health, Akita University School of Medicine.; 2 Department of Internal Medicine, Division of Cardiovascular Medicine, Akita University School of Medicine.

**Keywords:** sense of purpose in life, *ikigai*, cardiovascular diseases, mortality

## Abstract

**Background:**

Many studies have focused on disease causality, but few of them deal with health-promoting factors. Thus, we examined the effect of having a sense of purpose in life (*ikigai*) on mortality from cardiovascular disease (CVD).

**Methods:**

In 1988, we conducted a prospective cohort study of 2,959 Japanese subjects, ranging in age from 40 to 74 years, and followed them till the end of 2003. The level of their sense of purpose in life was evaluated by a self-administered questionnaire. After excluding those with a history of heart disease, stroke, or malignant tumor, 1,618 subjects (832 men and 786 women) who had completed the questionnaire were used in the analyses with Cox’s proportional hazards model.

**Results:**

During the average 13.3 years of follow up, 249 deaths (172 men and 77 women) occurred as a result of all causes: 32 from heart disease, 31 from stroke, 63 from CVD, and 104 from malignant tumors. The adjusted hazard ratios for death in men with a strong sense of purpose in life, as compared with those with a low sense of purpose, were 0.28 (95% confidence interval: 0.10-0.84) for stroke, 0.56 (0.28-1.10) for CVD, and 0.62 (0.45-0.86) as a result of all causes. In women, no significant relationship was found between having a sense of purpose in life and mortality; this was possibly because the smaller number of deaths reduced the statistical significance.

**Conclusion:**

We found that in men, having a sense of purpose in life affected the risk of death as a result of all causes, stroke, and CVD.

## INTRODUCTION

Many studies have suggested the association of mental stress with an increased risk of cardiovascular disease (CVD). Kranz et al^1^ reported that the physical and mental activities (involving emotions) of daily life could serve as triggers of ischemia in coronary artery disease (CAD) patients. Another study evaluated 196 patients enrolled in the Psychophysiological Investigations of Myocardial Ischemia Study, and found that ischemia induced by mental stress was associated with an almost 3-fold increase in the mortality rate of patients with CAD.^2^ Based on the data from The Japan Collaborative Cohort Study for Evaluation of Cancer Risk Sponsored by Monbusho (JACC Study), Iso et al^3^ reported that perceived stress levels were associated with increased mortality from stroke in women and increased mortality from CAD in men and women. They used psychosocial factors as the covariant in the analysis to evaluate the association between the risk of mortality and perceived stress, but they did not comment on the effect of psychosocial factors on the risk of mortality. Another study reported a relationship between the severity of heart failure and quality of life.^4^ Rozanski et al^5^ indicated that interventional trials with CVD patients, designed to reduce psychosocial stress, have been limited in both size and number.

To date, many studies focusing on disease causality, including perceived mental stress, have been reported, but few deal with the factors that promote health in the general Japanese population. One factor is having a sense of purpose in life (in Japanese, *ikigai*). This sense of purpose has been proposed to be a uniquely Japanese concept that has a positive effect on one’s life and makes one’s life worth living.^6^ In addition to investigating disease causality, elucidating the factors that promote health is important. We hypothesized that having a sense of purpose in life may help people maintain good health and reduce the risk of mortality. Thus, we examined the effect of having a sense of purpose in life on the risk of death from CVD.

## METHODS

In 1988, 2,959 people (1,306 men and 1,653 women) ranging in age from 40 to 74 years, who lived in O-town, Akita Prefecture in northern Japan, were enrolled in this study. At baseline, the participants underwent multiple health-screening examinations and completed a self-administered questionnaire regarding their lifestyles, including any previous history of myocardial infarction, stroke, or malignant tumors. Informed consent was obtained from the participants when they completed the questionnaire. The study population represented part of the population enrolled in the JACC Study, and this study was conducted in accordance with a data-use agreement. Details of the JACC Study have been reported elsewhere.^7^

The level of the sense of purpose experienced by people was measured based on their response (“definitely,” “yes,” “fairly,” or “no”) to the question, “Do you experience a sense of purpose in your daily life?” The perceived stress level was measured based on their response (“extremely high,” “high,” “medium,” or “low”) to the question, “What is the level of stress that you experience in your daily life?”

Those with a history of heart disease, including myocardial infarction (n = 41), cardiomyopathy (n = 3), chronic rheumatic heart disease (n = 12), stroke (n = 60), and/or malignant tumors (n = 19) were excluded from the analysis. Finally, the data of 1,618 (832 men and 786 women) people who completed the questionnaire were included in the analysis.

For mortality surveillance, we systematically reviewed death certificates, all of which were filed in the public health center in the locality of the subjects’ residence. The mortality data were dispatched to the Central Ministry of Health and Welfare, and the underlying causes of death were coded for National Vital Statistics, according to the International Classification of Diseases, Ninth Revision (ICD-9), for deaths between 1988 and 1994 and Tenth Revision (ICD-10) for deaths between 1995 and 2003. The code numbers for each cause of death were as follows: 391-398, 410-417, and 420-429 in ICD-9 and I01-02, I05-09, I20-28, and I30-52 in ICD-10 for heart disease; 430-438 in ICD-9 and I60-69 in ICD-10 for stroke; and 140-208 in ICD-9 and C00-97 in ICD-10 for malignant tumors. Deaths due to CVD were defined as the sum of deaths from heart disease and stroke.

Each participant was followed up from the date of completing the baseline questionnaire in December 1988 through December 2003, till the time of death, or till the participant moved out of the study area, whichever occurred first. The total follow-up period was 22,737 person-years.

We categorized participants according to their response to the question regarding their awareness of a sense of purpose in their life: those who responded with “definitely” (10.3% of the men and 8.7% of the women) and “yes” (32.3% of the men and 27.2% of the women) were categorized as having a strong sense of purpose in life. Those who responded with “fairly” (50.2% of the men and 55.6% of the women) and “no” (7.1% of the men and 8.5% of the women) were categorized as having experienced a low sense of purpose in life. The patients were also divided into 2 categories based on their responses to the question regarding their awareness of perceived stress: those who responded with “extremely high” (7.9% of the men and 8.4% of the women) and “high” (12.0% of the men and 13.1% of the women) were categorized as having a high level of perceived stress. Those who responded with “medium” (67.7% of the men and 70.4% of the women) and “low” (12.4% of the men and 8.1% of the women) were categorized as having a low level of perceived stress.

The means and proportions of the selected risk factors and perceived stress levels were calculated for the 2 categories related to the level of the sense of purpose experienced in life. A history of hypertension or diabetes, high body-mass index (BMI), smoking habit (current smokers or nonsmokers), alcohol consumption status (those who drank almost daily and those with a daily consumption level of more than 46 g of ethanol were categorized as “drinkers,” those who abstained from alcohol and former alcoholics were classified as “nondrinkers,” and the others were classified as “occasional drinkers”), the number of hours spent walking (walking for over 30 min/day or less), and occupation (full-time workers or others) were treated as potential influential factors. The age categories were 40-49, 50-59, 60-69, and 70-74 years. For the analysis of heart disease only, we used 3 age categories (40-59, 60-69, and 70-74 years) because none of the participants in their 40s died of heart disease.

The age-adjusted and multivariate-adjusted hazard ratios for those with a strong sense of purpose in life and their 95% confidence intervals (CI) were calculated for mortality from stroke, heart disease, CVD, malignant tumors, and all causes, after adjustment for potential confounding factors, using Cox’s proportional hazards model. These confounding variables composed of a history of hypertension, a history of diabetes, smoking, and perceived stress. The numbers of censored cases before the earliest event in a stratum, which were automatically excluded in the recurrence analysis of the Cox proportional hazards model, were as follows: 7 men and 21 women for stroke, 10 men and 2 women for heart disease, 7 men and 2 women for CVD, and 1 man for malignant tumor. The results were calculated separately for men and women. Differences between groups were determined by the Student’s *t* test or chi-square analysis, as appropriate. The significance level was set at *P* < 0.05. Statistical analyses were conducted using SPSS^®^ (version 11.5).

## RESULTS

During the average 13.3 years of follow up, 249 (172 men and 77 women) deaths occurred as a result of all causes, including 63 (41 men and 22 women) from CVD (32 [18 men and 14 women] from heart disease and 31 [23 men and 8 women] from stroke) and 104 (73 men and 31 women) from malignant tumors. The deaths resulting from heart disease consisted of 11 (5 men and 6 women) from heart failure, 7 (4 men and 3 women) from ischemic heart disease, and 14 (9 men and 5 women) from other types of heart diseases. The deaths occurring as a result of stroke consisted of 7 (6 men and 1 woman) from intracerebral hemorrhages, 7 (4 men and 3 women) from subarachnoid hemorrhages, and 17 (13 men and 4 women) from cerebral infarction.

Table 1 shows the gender-specific baseline characteristics of cardiovascular risk factors and perceived stress, according to the level of the sense of purpose experienced by the subjects. A strong sense of purpose was reported by 42.6% of the men and 35.9% of the women. As compared with men who reported a low sense of purpose, those with a strong sense of purpose had a lower frequency of a history of hypertension (*P* = 0.004). The relationship between having a sense of purpose in life and perceived stress was not statistically significant in men or women (*P* = 0.464 for men and *P* = 0.805 for women).

**Table 1.** Gender-specific mean value or prevalence of risk factors at baseline, based on the level of the sense of purpose experienced by the subjects.

Level of the sense of purpose in life	Men			Women		
	
	Low n = 477	High n = 355	*P* value	Low n = 504	High n = 282	*P* value
Average age ± SD (years)	55.2 ± 9.1	55.5 ± 9.1	0.775	56.0 ± 9.1	55.6 ± 9.7	0.580
History of hypertension (%)	18.9	11.5	0.004	14.7	13.5	0.642
History of diabetes (%)	3.1	3.4	0.850	2.8	3.9	0.390
Smokers (%)	75.3	69.6	0.068	3.6	1.4	0.079
High level of perceived stress (%)	19.1	21.2	0.464	21.2	22.0	0.805
Average BMI ± SD (kg/m^2^)	22.7 ± 2.5	23.2 ± 3.9	0.026	23.4 ± 3.1	23.3 ± 2.8	0.753
Alcohol consumers (%)	71.2	67.0	0.207	4.4	3.0	0.334
Hours spent walking (over 30 min/day) (%)	93.6	93.6	0.967	92.9	93.8	0.664
Occupation (full-time workers) (%)	74.6	79.1	0.132	35.3	39.4	0.250

The differences between groups were determined by the Student’s *t* test or chi-square analysis.

The numbers of missing values were as follows: for BMI, 31 men and 29 women with a low sense of purpose in life and 13 men and 9 women with a strong sense of purpose; for alcohol consumption, 33 men and 44 women with a low sense of purpose, and 19 men and 19 women with a strong sense of purpose; for the number of hours spent walking, 21 men and 22 women with a low sense of purpose, and 13 men and 9 women with a strong sense of purpose; for occupation, 29 men and 19 women with a low sense of purpose, and 10 men and 3 women with a strong sense of purpose.

SD: standard deviation; BMI: body mass index

Table 2 shows gender-specific age-adjusted and multivariate-adjusted hazard ratios and 95% CI for mortality from stroke, heart disease, CVD, malignant tumors, and all causes, using Cox’s proportional hazards model. Compared to men with a low sense of purpose in life, men with a strong sense of purpose had a significantly lower age-adjusted mortality from stroke, CVD, and all causes. Among men, significant multivariate-adjusted hazard risk (95% CI) after adjustment for potential risk factors other than perceived stress was as follows: 0.28 (0.10-0.84) for stroke and 0.62 (0.45-0.86) for all causes. After further adjustment for perceived stress, these differences were still statistically significant. The same trend was found for death from CVD in men, i.e., the multivariate-adjusted hazard was 0.56 (0.28-1.10). Among women, no significant relationship was found between the level of the sense of purpose and mortality from stroke, heart disease, CVD, or malignant tumors.

**Table 2.** The hazard ratios (HRs) and their 95% confidence intervals for deaths from stroke, heart disease, cardiovascular disease, malignant tumors, and all causes, based on the level of a sense of purpose in life.

Level of a sense of purpose in life	Men			Women		
	Low	High		Low	High	
	
	n = 477	n = 355	*P* value	n = 504	n = 282	*P* value
Stroke						
Number of deaths	19	4		4	4	
Age-adjusted HR	1	0.25 (0.09-0.74)	0.012	1	1.40 (0.35-5.63)	0.640
Multivariate-adjusted HR(1)	1	0.28 (0.10-0.84)	0.023	1	1.09 (0.26-4.62)	0.905
Multivariate-adjusted HR(2)	1	0.28 (0.10-0.84)	0.023	1	1.15 (0.27-4.88)	0.852

Heart disease						
Number of deaths	10	8		9	5	
Age-adjusted HR	1	0.86 (0.33-2.16)	0.732	1	0.93 (0.31-2.77)	0.889
Multivariate-adjusted HR(1)	1	1.10 (0.43-2.84)	0.844	1	0.98 (0.32-3.02)	0.979
Multivariate-adjusted HR(2)	1	1.09 (0.42-2.81)	0.861	1	1.02 (0.33-3.14)	0.967

Cardiovascular disease						
Number of deaths	29	12		13	9	
Age-adjusted HR	1	0.47 (0.24-0.92)	0.029	1	1.04 (0.44-2.44)	0.936
Multivariate-adjusted HR(1)	1	0.56 (0.28-1.10)	0.093	1	0.99 (0.40-2.42)	0.980
Multivariate-adjusted HR(2)	1	0.56 (0.28-1.10)	0.093	1	1.04 (0.43-2.53)	0.931

Malignant tumors						
Number of deaths	41	32		21	10	
Age-adjusted HR	1	0.94 (0.59-1.50)	0.797	1	0.82 (0.39-1.76)	0.613
Multivariate-adjusted HR(1)	1	0.93 (0.58-1.48)	0.754	1	0.85 (0.39-1.84)	0.682
Multivariate-adjusted HR(2)	1	0.93 (0.58-1.48)	0.750	1	0.88 (0.41-1.90)	0.742

All causes						
Number of deaths	114	58		54	23	
Age-adjusted HR	1	0.59 (0.43-0.82)	0.001	1	0.71 (0.43-1.16)	0.166
Multivariate-adjusted HR(1)	1	0.62 (0.45-0.86)	0.004	1	0.72 (0.44-1.19)	0.201
Multivariate-adjusted HR(2)	1	0.62 (0.45-0.86)	0.004	1	0.74 (0.45-1.22)	0.232

The multivariate-adjusted HR(1) was adjusted for age, history of hypertension, history of diabetes, and smoking habit.

The multivariate-adjusted HR(2) was adjusted for age, history of hypertension, history of diabetes, smoking habit, and perceived stress.

## DISCUSSION

In the present study, we showed that the risk of mortality from stroke, CVD, and all causes was lower in men with a strong sense of purpose, as compared with men with a low sense of purpose.

Our findings suggest that the level of the sense of purpose, as a positive perceived factor, is predictive of mortality in men. Sakata et al^8^ also reported a relationship between the perceived factors and the risk of mortality from CVD. The current findings were similar to the previous ones.

Another positive perceived factor is life satisfaction. A study on the relationship between the level of life satisfaction and mortality was conducted in Finland.^9^ According to the Finnish Twin Cohort, the definition of life satisfaction included interest in life, happiness, general ease of living, and not feeling a sense of loneliness. Those who reported dissatisfaction with life at baseline showed higher mortality than those who reported satisfaction with life: this was especially observed in men and alcoholics, but not in women.

Several factors may explain the relationship between having a sense of purpose in life and mortality from CVD. Many studies have reported a relationship between several negative perceived factors and CVD. First, perceived stress has consistently been reported as a risk factor for CVD.^1^^,^^2^^,^^3^^,^^10^ The mechanisms underlying the relationship between perceived stress and the risk of CVD involve the sympathetic nervous system,^11^ vascular endothelial cell damage, inflammatory processes,^12^ platelet activation,^13^^,^^14^ and progression of atherosclerosis, all of which can induce ischemia, arrhythmias, and increased blood pressure,^11^ which in turn can promote the development of CVD. It is possible that perceived stress influences the relationship between having a sense of purpose in life and the risk of mortality from CVD. We determined the relationship between perceived stress and a sense of purpose in life by using a chi-square analysis. We found no significant relationship between having a sense of purpose in life and perceived stress (Table 1). In a multivariate analysis, even after further adjustment for perceived stress, in men, we still observed a statistically significant relationship between having a sense of purpose in life and mortality from stroke, CVD, and all causes. This suggests that having a sense of purpose in life may not be related to perceived stress.

Several studies have reported the relationships between self-reported poor health, which is another negative perceived factor, and CVD. Self-reported poor health was related to obesity, smoking, and physical inactivity.^15^ It has also been reported that people living in deprived neighborhoods were at a higher risk of obesity and a disadvantaged lifestyle, which could be further aggravated with increased smoking and physical inactivity.^16^^,^^17^ Another study reported that physical activity protected against poor health, regardless of an increase in BMI and smoking; habitual smokers or physically inactive people were associated with higher hazard ratios for all-cause mortality, than those who were reported to be in better health.^18^ Self-reported health status may influence the sense of purpose in life; therefore, factors related to self-reported health status, such as a sedentary lifestyle, socioeconomic status, or neighborhood environment, may explain the relationship between having a sense of purpose in life and mortality from CVD and all causes. Unfortunately, we did not assess these factors in our study.

The mechanism of the relationship between a sense of purpose and the risk of CVD has not been studied. One explanation may be that silent cerebral infarction may cause a loss of a sense of purpose in life. We found a higher prevalence of hypertension in men with a low sense of purpose than in men with a strong sense of purpose (Table 1). Hypertension has been reported to be a risk factor for silent cerebral infarction.^19^^,^^20^ Silent cerebral infarction has been related to depression in many studies.^21^ Depression is known to increase the risk of mortality from CVD. ^22^ However, we did not use computed tomography or magnetic resonance imaging (MRI) scanning to examine cerebral infarction, so we were unable to ascertain whether silent cerebral infarction influenced the sense of purpose.

In Table 2, we present the adjusted hazard ratios using the major confounding factors. We found a similar trend in the relationship between a sense of purpose in life and mortality in the multivariate analysis adjusted for all the factors in Table 1 (data not shown). The criteria for the diagnosis of the cause of death changed in 1995. Analysis after excluding male participants whose follow up was completed before the end of 1994 still showed a statistically significant relationship between the level of the sense of purpose and mortality from stroke, CVD, and all causes. For the data between 1988 and 1994, we were unable to complete the analysis due to a small number of deaths.

Our study had some limitations. First, we were unable to examine the relevant issues more reliably due to the small number of deaths, especially among women. This may have influenced the results and, thus, gender-related difference in the results must be interpreted cautiously. We were also unable to examine the influence of the sense of purpose on mortality from intracranial hemorrhage, cerebral infarction, ischemic heart disease, and congestive heart failure separately because of the small numbers of such deaths. Second, our study subjects were all from rural Japan, and they included many older adults; thus, they may not be representative of the greater Japanese population. Finally, although the questionnaire used in our study was the same as that used in the JACC Study, its reliability and validity have not been established to the same degree as other questionnaires, such as the Short Form 36.

Studies focusing on the sense of purpose as a health-promoting factor and examining its influence on the risk of mortality from CVD are limited. Our results indicate that having a strong sense of purpose in life reduced the risk of mortality from all causes, stroke, and CVD in men. We suggest that health-promoting approaches, such as enhancing the sense of purpose, may be useful in reducing the risk of mortality from CVD.
